# Growth of a Nipple Adenoma After Estrogen Replacement Therapy

**DOI:** 10.7759/cureus.50843

**Published:** 2023-12-20

**Authors:** Elena E Skaribas, Jaime Tschen

**Affiliations:** 1 Dermatology, McGovern Medical School, Houston, USA; 2 Dermatology, Saint Joseph Dermatopathology, Houston, USA

**Keywords:** menopausal hormone therapy, myoepithelial cells, menopause, estrogen replacement therapy, nipple adenoma

## Abstract

A nipple adenoma is an epithelial tumor of the lactiferous ducts, typically affecting women aged 50-60 years old. This case report discusses a 52-year-old woman who developed a papillary adenoma of the right nipple after initiating oral estrogen replacement therapy (ERT) for perimenopausal symptoms. A 4 mm punch biopsy and subsequent immunohistochemistry stain revealed the proliferation of ductal structures consistent with a papillary adenoma and tumor cells expressing estrogen receptors (ER) and progesterone receptors (PR). Despite their benign nature, nipple adenomas may exhibit alterations in immunophenotype, including ER and PR expression, which could lead to potential tumor growth in women undergoing these treatments. This case describes the first reported growth of a nipple adenoma in the context of estrogen replacement therapy, highlighting a potential risk of hormone therapy in promoting hyperproliferation of benign tumors such as nipple adenomas. When utilizing ERT, it is important to weigh the potential advantages and risks, as its application in the management of vasomotor symptoms during menopause may increase the risk of both breast cancer and benign proliferative breast diseases. These considerations underscore the need for individualized therapy when approaching perimenopausal and postmenopausal care.

## Introduction

A nipple adenoma is a rare, benign epithelial tumor of the lactiferous ducts that only affects a few women each year and has a peak age of onset occurring between 50-60 years of age [1,2]. Common presentation is a palpable mass of the nipple often associated with pain, ulceration, swelling, and discharge [1-4]. Histologically, nipple adenomas are characterized by the adenomatous proliferation of ducts lined by retained epithelial and myoepithelial cell layers with no cellular atypia [3].

Menopausal hormone therapy is a treatment used to alleviate severe vasomotor symptoms, which can affect up to 75% of all women during the onset of menopause [5,6]. Despite its efficacy, estrogen replacement poses significant risks and adverse effects that do not always outweigh the benefits of therapy [7]. Although randomized trials have linked estrogen therapy to an increased risk of breast cancer, the effect of hormone therapy on benign proliferative breast disease has not been well defined [8]. Because nipple adenomas are a rare condition, the etiology behind the development of these tumors is still unknown, and the condition itself has only been described through several case reports. The only reported associations with the development of this pathology have included pregnancy and concurrent carcinoma of the breast [9,10]. In this case report, a patient presented with a papillary adenoma of the nipple after undergoing oral estrogen replacement therapy for perimenopausal symptoms, making this the first case describing an association between estrogen replacement therapy and nipple adenoma growth. 

## Case presentation

**Figure 1:**
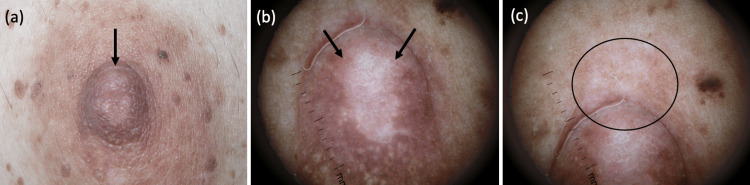
Nipple adenoma in a 52-year-old woman after oral estrogen replacement therapy (a) Appearance of the entire right nipple and surrounding areolar skin. Adenoma seen at the superior aspect of the nipple (arrow). (b) Dermatoscopic view of the same mass at the superior aspect of the right nipple (arrows). (c) Observed reticulated keratin with erythema at the margin of the nipple (circle).

We present the case of a 52-year-old woman who underwent oral estrogen replacement therapy due to perimenopausal symptoms six months after a hysterectomy and bilateral oophorectomy. Approximately one month after initiating medication therapy, she noted a growth on the right nipple (Figure 1).

**Figure 2:**
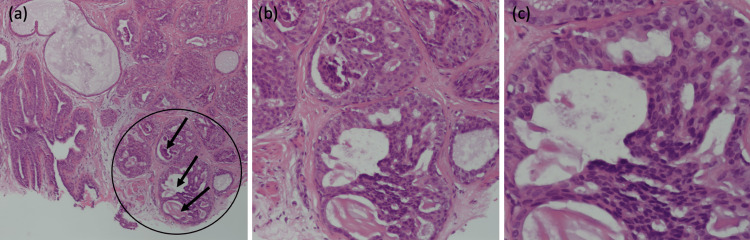
Histological section with H&E staining taken from 4 mm punch biopsy of the right nipple adenoma. Images show three magnifications of one lobe of the mammary gland, indicated by the circle. Images show benign adenomatous proliferation of epithelial cells of the lactiferous ducts (arrows). (a) Low power magnification 10x. (b) Low power magnification 50x. (c) High Power magnification 100x.

The patient presented to the gynecology clinic and was then referred to dermatology for further work-up. A physical exam of the patient revealed a small mass on the superior aspect of the right nipple, while the left breast was normal upon inspection. A 4 mm punch biopsy was taken, and the specimen revealed a proliferation of ductal structures with apocrine differentiation consistent with a papillary adenoma of the nipple (Figure 2).

**Figure 3:**
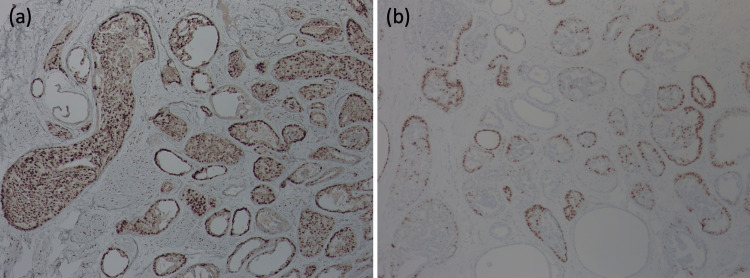
Immunohistochemical (IHC) study of right nipple biopsy. Brown IHC staining represents the targeted hormone positive cells. (a) 4+ estrogen receptor positive 10x. (b) 1+ progesterone receptor positive 10x.

An immunohistochemistry study further revealed that the tumor was positive for estrogen receptor and progesterone receptor markers (Figure 3). 

After the diagnosis of papillary adenoma of the nipple was determined, the patient discontinued estrogen therapy and underwent full excision of the lesion without complications. 

## Discussion

Among the various symptoms encountered during menopause, vasomotor symptoms are recognized as the most pervasive and incapacitating [5,6]. Hot flashes and night sweats affect the majority of perimenopausal women and can lead to both physical and social impairment. Studies have shown that patients experiencing symptoms of menopause face an increased risk of mood, anxiety, and sleep disorders [11]. Furthermore, among women aged 45-60 years old, those reporting higher severity of menopausal symptoms had increased adverse work outcomes, and overall workdays missed due to menopausal symptoms equated to an average annual loss of $1.8 billion in the United States [6].

Estrogen replacement therapy has been recognized for decades as the most effective treatment for vasomotor and vaginal symptoms associated with menopause; however, widespread use of hormone treatment declined in the early 2000s after randomized clinical trials suggested that estrogen and combined hormone therapies carried significant risks [7]. Results from the Women's Health Initiative (WHI) clinical trial published in 2002 concluded that the overall risk from the use of estrogen replacement therapy exceeded the benefits, with primary adverse outcomes including coronary heart disease and invasive breast cancer [12]. The use of hormone therapy in menopausal patients declined soon after these findings, with the use of estrogen replacement therapy falling from 22% in 2000 down to 5% in 2010 [13]. Despite this shift, the use of estrogen replacement therapy has gained new support in recent years as more research into hormone therapy has increased our understanding of its effects. A recent position statement by the North American Menopause Society (NAMS) argued that no single trial's findings should dictate the use of estrogen replacement therapy for all patients and agreed that women without contraindications could significantly benefit from therapy, especially if younger than 60 years old or less than 10 years from the onset of symptoms [14]. Subsequently, other major medical societies, including the American College of Obstetricians and Gynecologists (ACOG) and the American Association of Clinical Endocrinology (AACE), now deem estrogen replacement therapy an appropriate form of menopausal symptom management [15].

Estrogen and progesterone signaling via nuclear receptor binding are important in the growth of breast tissue, but estrogen replacement therapy during menopause could potentially cause hyperproliferation of tumors that have estrogen receptor and progesterone receptor expression. Despite being characterized as a benign lesion, prior studies of nipple adenomas have shown distinct alterations in the immunophenotype of epithelial and myoepithelial cell layers. For example, changes in the expression of certain myoepithelial markers such as CD10, p63, and M-actin were seen in nipple adenomas and other benign breast lesions compared to normal ductal tissue [4,16]. In previous case reports, changes in estrogen receptor and progesterone receptor immunoexpression were also reported in patients with nipple adenomas [17,18]. Although rare, it is important to take into account the possibility of benign tumor development, such as papillary adenomas of the nipple, when considering hormone therapy for perimenopausal women. Despite this potential risk, the association of estrogen replacement therapy and its effect on benign tumor growth of the breast, including nipple adenomas, requires further study in order to be applied in a clinical setting. Due to their rare nature, nipple adenomas have yet to be widely studied in larger sample sizes, which means confounding variables outside those suggested in case reports could also contribute to their growth.

## Conclusions

Given the continued widespread use of estrogen replacement therapy during menopause, it is essential to carefully weigh both the advantages and disadvantages associated with this treatment. While women who suffer from severe symptoms such as hot flashes and other vasomotor effects can gain significant relief from estrogen replacement therapy, it's crucial to acknowledge the potential for estrogen-sensitive tumor growth as a contraindication for these medications. Not only have past randomized trials linked estrogen therapy to an increased risk of breast cancer, but novel studies also suggest an association with benign proliferative breast disease. In this case report, a patient experienced the growth of an estrogen and progesterone-positive nipple adenoma after undergoing oral estrogen replacement therapy. While estrogen and progesterone signaling are key in the growth of breast tissue, hormone therapy may potentially contribute to the hyperproliferation of tumors that have estrogen receptor and progesterone receptor expression, as observed in this patient. Consequently, when contemplating hormone therapy for perimenopausal women, it is essential to take into account the risk of benign tumor development, such as papillary adenomas of the nipple. Because of this risk, the development of any new breast lesion while a patient is on estrogen therapy warrants a biopsy for further investigation. 
